# An Exploratory Analysis of Public Awareness and Perception of Ionizing Radiation and Guide to Public Health Practice in Vermont

**DOI:** 10.1155/2015/476495

**Published:** 2015-04-28

**Authors:** Katherine M. Evans, Jenna Bodmer, Bryce Edwards, James Levins, Amanda O'Meara, Merima Ruhotina, Richard Smith, Thomas Delaney, Razelle Hoffman-Contois, Linda Boccuzzo, Heidi Hales, Jan K. Carney

**Affiliations:** ^1^University of Vermont College of Medicine, 89 Beaumont Avenue, Burlington, VT 05405, USA; ^2^Vermont Department of Health, 108 Cherry Street, Burlington, VT 05402, USA

## Abstract

Exposure to ionizing radiation has potential for acute and chronic health effects. Within the general public of the United States, there may be a discrepancy between perceived and actual health risks. In conjunction with the Vermont Department of Health, a survey designed to assess public perception and knowledge of ionizing radiation was administered at 6 Vermont locations (*n* = 169). Descriptive and inferential statistical analyses were conducted. Eighty percent of respondents underestimated the contribution of medical imaging tests to total ionizing radiation exposure. Although only thirty-nine percent of participants were confident in their healthcare professional's knowledge of ionizing radiation, most would prefer to receive information from their healthcare professional. Only one-third of individuals who received a medical imaging test in the past year were educated by their healthcare professional about the risks of these tests. Those who tested their home for radon were twice as likely to choose radon as the greatest ionizing radiation risk to self. Although respondents had an above-average education level, there were many misperceptions of actual risks of exposure to ionizing radiation, particularly of medical imaging tests. Educating healthcare professionals would therefore have a profound and positive impact on public understanding of ionizing radiation.

## 1. Introduction

Ionizing radiation is a broad, complicated, and often misunderstood topic. Exposure to ionizing radiation is associated with both acute and chronic disease states, especially as the radiation dose increases [1–5]. Children are particularly susceptible to ionizing radiation and, because of their young age, may be more likely to experience delayed manifestations of ionizing radiation exposure [6]. Nevertheless, individuals are constantly exposed to ionizing radiation from a variety of sources: naturally occurring, medical imaging, and other human-made. Studies indicate a difference in both risk perception and knowledge of actual sources of ionizing radiation between the general public and radiation experts [7–12]. This is in part due to how health risks are portrayed by mass media, which may misinform the general public through exaggeration of some sources and minimization of others; the technical language of radiation risk assessment also plays a role, especially given educational discrepancies in the population at large [7–9, 11]. Indeed, perceived risk of nuclear power is strongly emotional and unlikely to be altered; the population generally perceives nuclear power as extremely risky and posing a much higher risk of exposure to the general public than in actuality [11, 13–15].

**Figure 1:**
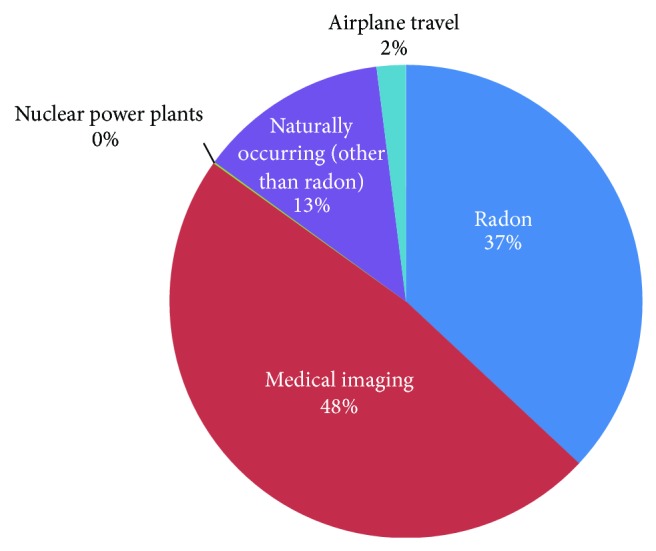
Actual ionizing radiation exposure for the average person in the USA [8].

Naturally occurring radon is perhaps one of the best known environmental sources of ionizing radiation [10, 16]. This element is ubiquitous, and high exposure to this gas has been consistently associated with increased risk for developing lung cancer [17–20]. Another major source of naturally occurring ionizing radiation is potassium-40 (K-40), a radioactive isotope; in fact, this common element represents the largest dose of annual exposure to ionizing radiation [21]. Both radon and K-40, while present in the environment, exist in varying concentrations according to geographic location, making one's exposure to such sources of ionizing radiation dependent upon one's place of residence and employment [21]. An additional factor for variation in annual ionizing radiation exposure between individuals is diagnostic and therapeutic medical procedures [2, 22–24]. Use of ionizing radiation-producing medical studies has boomed; from medical procedures alone, Americans were exposed to more than seven times more ionizing radiation in 2006 than in the early 1980s [22]. Currently, on a population level, medical imaging comprises nearly half of the annual ionizing radiation exposure (see Figure 1) [8].

Studies have suggested that the general public is not concerned about exposure to ionizing radiation from medical procedures because of a widespread notion that healthcare professionals have received extensive training in principles of radiation and are competent in minimizing risk [23, 25]. However, healthcare professionals may not be as informed as the public believes [23, 24]. Physicians tend to underestimate doses of ionizing radiation from medical sources, and some are even unaware of which medical tests are sources of ionizing radiation [25–27]. There exists a great need to educate not only the general public but also healthcare professionals, in basic concepts of ionizing radiation exposure and risk. Thereby, healthcare professionals become stewards of public health in providing accurate information to their patients. In order to determine an effective methodology for instituting educational programs, it is vital to first gain an appreciation for current knowledge and perceptions which exist about ionizing radiation among Vermonters.

## 2. Methods

This project was carried out after review and acceptance by the approval of the University of Vermont Research Protection's Office and under Instructor's Assurance, in conjunction with the Vermont Department of Health. Following a literature review, a 20-question survey was designed to assess public perception and knowledge of ionizing radiation. Demographic information on respondents included town of residence, length of time in current residence, if residence had been tested for radon, gender, age, education level, age of individuals in household, and if occupation was in healthcare and/or science. Respondents were asked to rank their confidence in their knowledge of ionizing radiation on a Likert-style scale ranging from 1 (not at all confident) to 5 (highly confident). Individuals who selected either 4 or 5 were considered to be “confident” in their knowledge of ionizing radiation, while those who selected 1 or 2 were considered to be “poorly confident.” In order to evaluate respondents' knowledge, they were asked to select potential sources of ionizing radiation from the following: computed tomography (CT) scan, magnetic resonance imaging (MRI), chest X-ray, mammogram, ultrasound, and dental X-ray. Of these, only CT scan, chest X-ray, mammogram, and dental X-ray expose patients to ionizing radiation. MRI and ultrasound employ principles of magnetic fields and sound waves, respectively. Selecting CT scan, chest X-ray, mammogram, and dental X-ray and not selecting MRI and ultrasound entailed a perfect score on this question. Respondents who correctly classified 5 or 6 of these medical imaging tests were considered to be “knowledgeable.”

Respondents were then asked if they knew if they had received a medical imaging test that uses ionizing radiation and, if so, if they had been counseled on the risks and benefits of the imaging study by their healthcare professional. Using the previously described Likert-style scale, respondents ranked their confidence in their healthcare professional's knowledge on medical imaging tests involving ionizing radiation.

In order to compare perceived health risk of various sources of ionizing radiation, respondents were asked to select which of the following posed the greatest and least health risk to both the respondent and the average Vermonter: medical imaging tests that use ionizing radiation, radon, other natural sources of ionizing radiation, nuclear power plants, or airplane travel.

Finally, respondents were asked to report where they received information about ionizing radiation, which source(s) were most trusted to provide information about ionizing radiation, and where they preferred to receive information about ionizing radiation.

This survey was administered at six Vermont locations (*n* = 169) across Orleans, Chittenden, and Windham counties, chosen based on access to large community events. An additional 24 responses were gathered from the State of Vermont Radiological Sampling Team (RST) and were used as an expert reference group and statistically analyzed separately. Demographic data of respondents was organized in tabular format (see Table 1). Twenty percent of the surveys were randomly selected for a quality control check. Descriptive and inferential statistical analyses were conducted using SPSS and statistical significance was determined using Mantel-Haenszel odds ratios with 95% confidence intervals. Alpha was set at 0.05 (two-tailed) for all statistical analyses.

## 3. Results

### 3.1. Medical Sources of Ionizing Radiation

**Figure 2:**
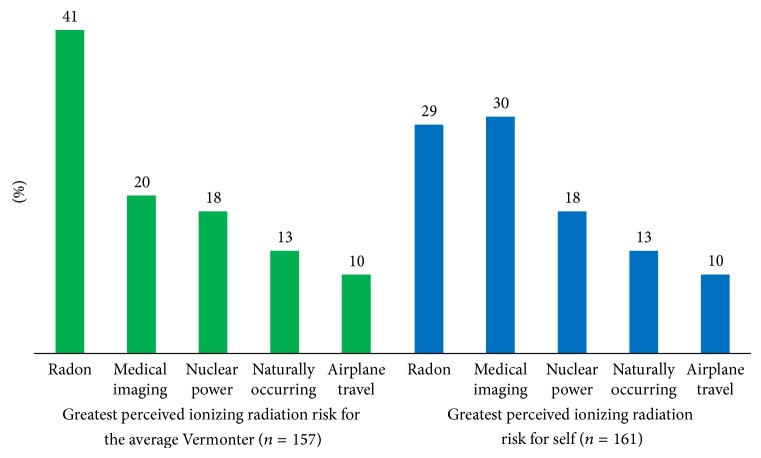
Greatest perceived ionizing radiation risk to the average Vermonter versus risk to self, based on responses from the general Vermont population.

Respondents who were “knowledgeable” about medical sources of ionizing radiation tended to have higher education level (OR = 3.38, CI 95% 1.38–8.35, and *p* = 0.008) and were more likely to work in science/healthcare (OR = 5.44, CI 95% 2.32–12.79, and *p* < 0.001). Eighty percent of respondents underestimated the contribution of medical imaging tests to total population ionizing radiation exposure (Figure 2). Overall, respondent results were incongruent with the actual distribution of radiation sources (Figures 1-2).

### 3.2. Radon as a Source of Ionizing Radiation

Men were more than women likely to select radon as being the greatest risk to average Vermonters (OR = 2.07, CI 95% 1.07–3.99, and *p* = 0.03). Those that had their home tested for radon were more than three times as likely to choose radon as the greatest risk of ionizing radiation to self (OR = 3.20, CI 95% 1.47–6.97, and *p* = 0.003) and there was a trend in which participants who had home radon tests were more likely to indicate radon as the greatest risk of ionizing radiation to Vermonters (OR = 2.07, CI 95% = 0.97–4.36, and *p* = 0.06).

### 3.3. Nuclear Power as a Source of Ionizing Radiation

Respondents with higher education level (OR = 2.86, CI 95% 1.15–7.14, and *p* = 0.024) and males (OR = 4.04, CI 95% 1.71–9.52, and *p* = 0.001) were more likely to correctly select nuclear power as the least risk for Vermonters. There was a nonsignificant trend towards participants of younger age being more likely to correctly identify nuclear power as posing the least risk to Vermonters (OR = 2.91, CI 95% 0.83–10.23, and *p* = 0.096).

### 3.4. Role of Healthcare Professionals

Respondents with higher education level (OR = 2.86, CI 95% 1.15–7.14, and *p* = 0.024) and males (OR = 4.04, CI 95% 1.71–9.52, and *p* = 0.001) were more likely to correctly select nuclear power as the least risk for Vermonters. There was a nonsignificant trend towards participants of younger age being more likely to correctly identify nuclear power as posing the least risk to Vermonters (OR = 2.91, CI 95% 0.83–10.23, and *p* = 0.096).

### 3.5. Vermont Radiological Sampling Team (RST)

Members of the Vermont RST were more likely to be “knowledgeable” about ionizing radiation (OR = 27.15, CI 95% 7.63–96.57, and *p* < 0.001) than non-RST respondents. While 37% of the RST were “confident” in their knowledge of ionizing radiation compared to 8% of the non-RST respondents, this relationship was not statistically significant (OR = 0.51, CI 95% 0.21–1.23, and *p* = 0.13). Specifically, RST individuals were more likely to correctly identify nuclear power as being the lowest source of exposure to the general public (OR = 3.70, CI 95% 1.51–9.05, and *p* = 0.004) and radon as the highest (OR = 2.55, CI 95% 1.07–6.12, and *p* = 0.035) ionizing radiation sources overall, as compared to the non-RST respondents. RST members were also more likely than the general Vermont population to prefer to receive information regarding ionizing radiation from a scientific publication (OR = 3.25, CI 95% 1.31–8.04, and *p* = 0.011).

## 4. Discussion

Medical imaging constitutes the largest source of radiation exposure; natural sources, such as radon, comprise the second largest source [8]. However, our exploratory analysis revealed considerable misunderstanding of sources of ionizing radiation by the general Vermont population (Figures 1-2). This echoes the findings of many other studies and illuminates a perpetuated lack of understanding among the general public [7–9, 16, 28, 29]. Even more striking in our study was the education level of respondents, the majority of whom had reached an above-average education level [30]. Current methods of disseminating information concerning ionizing radiation are clearly suboptimal and are of public health concern.

The Vermont Department of Health (VDH) has extensively publicized the risk of radon and held public campaigns encouraging residents to have their living environment tested. Our study revealed that these efforts may have been successful, largely in educating about radon and its potential health effects. Many of our respondents had tested their living environment for radon, perhaps as a direct result of the VDH's efforts to heighten awareness. Respondents who tested their home for radon were more likely to rank radon as their greatest source of ionizing radiation exposure, indicating that while their knowledge base was lacking, they had gleaned a deeper understanding of radon. In comparison, participants who have not tested their living environment for radon may not have been aware of the VDH campaign and did not rank radon as being a top source of ionizing radiation. This highlights the success of the VDH in providing public education, suggesting that pursuing other public health campaigns would be equally efficacious. Indeed, targeting healthcare professionals would likely be especially fruitful, as these individuals are able to perpetuate such education among all of their patients.

The majority of respondents indicated that they would prefer to receive information about ionizing radiation from their healthcare professional, but many did not express having confidence in their healthcare professional's knowledge of the subject. Indeed, less than one-third of individuals received any sort of education from their health care professional before undergoing a medical imaging test involving ionizing radiation [2, 23, 24]. Given not only the increasing utilization of medical imaging tests, [2, 23] but also the unique role of healthcare professionals in teaching their patients, it is essential that medical workers be effectively equipped with knowledge regarding ionizing radiation. It is clear that this goal is far from being achieved. Respondents who worked in science/healthcare reported their confidence in their knowledge as being poor. Topics of ionizing radiation are seldom, if ever, included in medical education [31]. Thus, medical education curricula may be an integral component for public health outreach. Educating health care professionals and students would enable these individuals to help teach their patients about ionizing radiation, particularly as it relates to medical imaging as compared to other sources.

The Vermont RST is comprised of volunteers who are recruited from the VDH, Vermont Agency of Natural Resources, Vermont Department of Labor, and the Vermont Agency of Agriculture, Food, and Markets. These individuals possess a wide range of educational and employment backgrounds; very few have any prior expertise on ionizing radiation. Every year, members of the Vermont RST participate in four training seminars not only on principle and theory of ionizing radiation, but also on how to safely collect samples for testing. The efficacy of such training was evidenced in the results of our survey. Respondents from the Vermont RST demonstrated a higher knowledge base regarding ionizing radiation than the general Vermont population. They also tended to prefer to receive information regarding ionizing radiation from a scientific publication rather than from a healthcare professional. This likely reflects RST's understanding of misperceptions of ionizing radiation and ability to identify experts in the field. A pilot program for educational awareness can be modeled from the methods used to educate the RST.

The present study is limited by a relatively small sample size. Our intent was to conduct an exploratory analysis for the entire state of Vermont; however, survey respondents were largely from northwestern Vermont, and as other geographic locations were less represented our findings may not be generalizable to all Vermont residents. Nevertheless, this potential limitation suggests that, even among our highly educated group of respondents, there is a substantial need to educate Vermonters about ionizing radiation.

## 5. Conclusions

Only eight percent of respondents from the general public in four Vermont counties expressed having confidence in their knowledge of ionizing radiation, indicating a great need for additional public education. As the majority of respondents prefer to receive information from their healthcare professional and given the continually increasing utilization of medical imaging tests using ionizing radiation, educating current and future healthcare professionals would have a profound and positive impact on public awareness of ionizing radiation.

## Figures and Tables

**Table 1:** Demographics of respondents.

Population	Gender (%)		Age (%)			Education (%)				Occupation (%)	
	Male	Female	18–45	46–65	66+	High school	Some college	College	Graduate degree	Healthcare or science	Other
General Vermont (169)	43.2	56.8	75.74	18.93	4.14	11.24	31.36	42.01	15.38	25.44^∗^	72.19^∗^
Radiation Sampling Team (24)	83.33	16.67	33.33	62.5	4.17	4.17	4.17	54.17	37.5	75.0	25.0

^∗^Note: four respondents did not answer this question and thus were not included in either occupational designation.
